# A systematic review of recommendations on screening strategies for breast cancer due to hereditary predisposition: Who, When, and How?

**DOI:** 10.1002/cam4.3898

**Published:** 2021-05-01

**Authors:** Yitong Cai, Jiang Li, Ya Gao, Kelu Yang, Jie He, Ni Li, Jinhui Tian

**Affiliations:** ^1^ Evidence‐Based Center Lanzhou University Lanzhou China; ^2^ National Cancer Center/National Clinical Research Center for Cancer/Cancer Hospital Chinese Academy of Medical Sciences and Peking Union Medical College Beijing China; ^3^ Evidence‐Based Medicine Center School of Basic Medical Sciences Lanzhou University Lanzhou China

**Keywords:** AGREE II, breast cancer, genetic mutated, guideline, screening

## Abstract

**Background:**

Breast cancer is a global health problem that cannot be underestimated. Many studies have shown that breast cancer is related to pathogenic mutations in hereditary predisposition genes. Clinical practice guidelines play a vital role in guiding the selection of breast cancer screening. Little is known about the quality and consistency of guidelines’ recommendations and their changes over these years.

**Methods:**

We reviewed the existing screening guidelines for genetic susceptibility to breast cancer and assessed the methodological quality, and summarized the recommendations to aid clinicians to make decisions. We conducted a systematic search in PubMed, Embase, Web of Science, and guideline‐specific databases, aiming to find the guidelines of breast cancer due to hereditary predisposition. The necessary information was exacted by Excel. We also summarized different evidence grading systems. The qualities of the guidelines were assessed by the Appraisal of Guidelines Research and Evaluation II (AGREE II) instrument.

**Results:**

A total of 54 recommendations from 13 guidelines were extracted. Generally speaking, the recommendations were consistent, mainly focusing on mammography and MRI.

**Conclusions:**

The recommendations differ in details. Moreover, different guidelines are based on different grading systems, and some guidelines are not divided for age limits, which may limit the promotion and implementation of the guidelines. It is suggested that improvement can be made in this regard in the future.

## Graphical abstract

Systematic and comprehensive search of breast cancer gene mutation screening guidelines, and summarize and analyze the recommendations, to find out the current problems

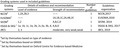

## 1 BACKGROUND

Breast cancer is a serious public health problem and the leading cause of death among women.^1^, ^2^ According to the latest statistics from the World Health Organization (WHO), breast cancer accounts for 10% of all types of cancers and 6.5% of global mortality.^3^ In terms of mortality, morbidity, psychological pressure, and economic costs, breast cancer caused huge social burden.^4^, ^5^ Over the past 25 years, investments in screening and other interventions have reduced cancer mortality by 27%.^6^ It has been proved that breast cancer screening is the most effective way to improve the survival rate and life quality of patients with breast cancer.^7^ But breast cancer screening has been a controversial issue for decades.^8^

In recent years, the prevalence of breast cancer has gradually increased, and the occurrence of breast cancer has been confirmed to be related to pathogenic mutations in hereditary predisposition genes.^9^, ^10^ One of the most crucial factors in the management of breast cancer is genetics.^11^ Among the gene mutations associated with breast cancer, the main gene refers to BRCA1/2.^12^ In addition to BRCA1 and BRCA2, at least seven other genes [ATM,^13^, ^14^, ^15^ CDH1,^16^, ^17^ CHEK2,^18^, ^19^ NF1,^20^, ^21^ PALB2,^22^, ^23^ PTEN,^24^, ^25^ and TP53^26^, ^27^ ] are associated with the risk of breast cancer (https://www.genecards.org/Search/Keyword?queryString=breast%20cancer), which has been used to provide information for breast cancer risk management.

Guidelines are widely accepted as necessary tools that transfer evidence into practice, thus enhancing clinicians’ and patients’ decisions, decreasing cost, and avoiding harm.^28^ Currently, there have been many guidelines for the screening of breast cancer due to hereditary predisposition, and different guidelines may have different recommendations.^29^ Previous studies on international health‐care systems have shown gaps in the implementation of population‐based screening of pathogenic mutations in hereditary breast cancer predisposition genes, and it is imperative to improve health‐care providers’ understanding of existing recommendations for the screening of pathogenic mutations in hereditary breast cancer predisposition genes.^30^, ^31^ Given recent developments in the management of mutated breast cancer and the importance of understanding the differences among global recommendations, we systematically indexed existing relative guidelines and summarized corresponding recommendations for pathogenic mutations in hereditary breast cancer predisposition genes, so as to provide references for clinical workers.

## 2 METHODS

### 2.1 Data sources and selection criteria

A systematic search in PubMed, Embase, Web of Science, and the Cochrane library was set up on 10 July 2020. At the same time, we also searched the following guideline databases: the National Guidelines Clearinghouse (NGC), the Guidelines International Network (G‐I‐N), the National Institute for Health and Care Excellence (NICE), the China Guideline Clearinghouse (CGC), and the Scottish Intercollegiate Network (SIGN). Meanwhile, the following related websites were also searched: the World Health Organization (WHO) website, National Comprehensive Cancer Network (NCCN) website, American Society of Clinical Oncology (ASCO) website, U.S. Preventive Services Task Force (USPSTF) website, UP‐TO‐Date website, and BMJ Best Practice. References of each guideline were also reviewed.

Guidelines we finally include must meet the following criteria: (1) Research types are published guidelines and (2) Containing recommendations on breast cancer screening are due to hereditary predisposition. The exclusion criteria are as follows: summary of guidelines, interpreted versions of guidelines, draft guidelines, non‐English guidelines, and old versions of the updated guidelines.

### 2.2 Study selection and data extraction

Two reviewers, JL and YTC, who have studied evidence‐based medicine, independently screened the records according to the inclusion and exclusion criteria and cross‐checked. If no consensus is reached, the disagreement is resolved through discussion or by a third reviewer JHT.

Reviewers YG and KLY extracted information using a predesigned extraction sheet. The extracted content includes publication time, organization, the country of the guideline, and whether the guideline is an updated version, and whether the guideline development team includes radiologists, funding, search year, and grading systems. We summarized the recommendations in the Table according to “who," "when," and “how". Meanwhile, the evidence basis for the recommendations, the level of evidence, and the strength of recommendations were also extracted.

### 2.3 Assessment of guideline quality

The Appraisal of Guidelines Research and Evaluation II (AGREE II) instrument was used to evaluate eligible guidelines’ methodological quality. The AGREE II instrument is the new international tool to assess the methodological quality of guidelines. It includes 23 items and six domains: Scope and purpose (items 1 to 3), Stakeholder involvement (items 4 to 6), Rigor of development (items 7 to 14), Clarity of presentation (items 15 to 17), Applicability (items 18 to 21), and Editorial independence (items 22 and 23) (Retrieved from https://www.agreetrust.org.). Each item is rated on the scale of 1 to 7, and 1 indicates strong disagreement and 7 indicates complete agreement.^32^

Four researchers, YTC, JL, YG, and KLY, who have been trained in the evaluation of guidelines independently, evaluated the guidelines’ items, and then calculated the percentage score of the domains based on the AGREE II instrument as follows: (obtained score—lowest score)/(highest score—lowest score).^31^ Finally, we divided the guidelines into three categories: Recommended scores should be over 60, modified recommended scores are 30–60, and not recommended scores are less than 30.^33^

### 2.4 Data synthesis and analysis

We summarized the characteristics, grading systems, and details of recommendations. For each eligible guideline, the AGREE II score of each domain and each overall score were represented by mean and standard deviation (SD). The internal correlation coefficient (ICC) is one of the indexes, measuring interobserver reliability and test–retest reliability. The ICCs were calculated based on the results, and the reliability and measurement consistency were evaluated. ICC is obtained by dividing individual variability, so its value is between 0 and 1. Meanwhile, 0 means that it is not trusted, and 1 means that it is completely trustworthy. The degree of agreement 0.01–0.20 is poor, 0.21–0.40 is fair, 0.41–0.60 is moderate, 0.61–0.80 is considerable, and 0.81–1.00 is very good.^34^ The analysis was conduct by SPSS 19.0 software (SPSS Inc).

## 3 RESULTS

**FIGURE 1:**
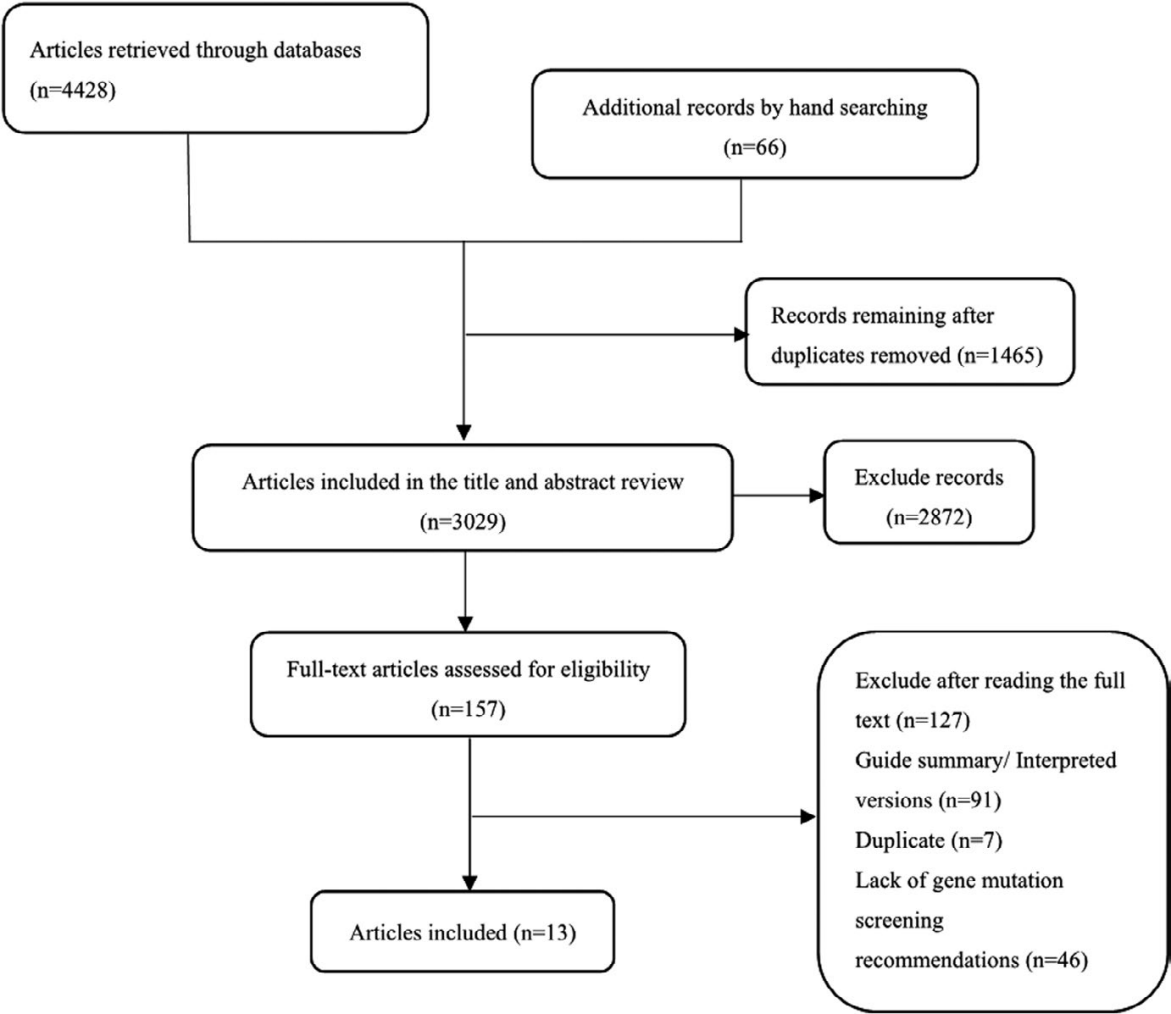
Summary of evidence search and selection

Our literature search identified 4494 guidelines, after excluding duplicates, and according to our inclusion and exclusion criteria, 13 were proved eligible in this review. The detailed search results are shown in Figure 1.

### 3.1 General characteristics

Table 1 displays the general characteristics of the included guidelines. Those eligible 13 guidelines^35^, ^36^, ^37^, ^38^, ^39^, ^40^, ^41^, ^42^, ^43^, ^44^, ^45^, ^46^, ^47^ from nine countries were produced by 13 agencies published between 2007 and 2019, and included eight updates.^35^, ^36^, ^37^, ^38^, ^44^, ^45^, ^46^, ^47^ Most of the guidelines are drawn from Europe, and the United States guidelines account for the largest proportion, reaching 23.08%.^38^, ^42^, ^45^ There are two (15.38%) in the United Kingdom.^35^, ^37^ EUSOMA guideline is a general European guideline. Canada,^36^ New Zealand,^39^ China, Hong Kong,^40^ Spain,^41^ Switzerland,^43^ Japan,^46^ and Germany^47^ have only one breast cancer screening guideline for gene mutations. Only one guideline indicated the source of funding.^36^ Six guidelines (46.15%) clearly stated that the guideline development group included radiologists. All guidelines reported less on systematical searching, and only two guidelines (15.38%) reported systematic search processes, but none of the guidelines reported the search year.

**TABLE 1:** Characteristics of 30 included guidelines on screening for breast cancer

Guideline organization, Year (Reference)	Country	Version	Systematical search	Search year	Funding	Is there any radiologists involved
NICE,2019	UK	Updated	Y	NR	NR	NR
CCO, 2018	Canada	Updated	NR	NR	Y^a^	NR
TRCR, 2019	UK	Updated	NR	NR	NR	NR
NCCN,2017	USA	Updated	NR	NR	NR	NR
ARG,2007	New Zealand	Original	NR	NR	NR	Y
CEWG,2018	China, HongKong	Original	NR	NR	NR	Y
SEOM, 2014	Spain	Original	NR	NR	NR	N
ACR, 2018	USA	Original	NR	NR	NR	Y
ESMO,2016	Switzerland	Original	NR	NR	NR	NR
EUSOMA,2017	European	Updated	NR	NR	NR	NR
ACS,2007	USA	Updated	NR	NR	NR	Y
JBCS, 2019	Japan	Updated	NR	NR	NR	Y
DGGG & DKG, 2017	Germany	Updated	Y	NR	NR	Y

Abbreviations: ACR, American College of Radiology; ACS, American Cancer Society; ARG, Auckland Radiology Group; CCO, Cancer Care Ontario; CEWG, Cancer Expert Working Group on Cancer Prevention and Screening; DGGG & DKG, German Society for Gynecology and Obstetrics and the German Cancer SocietyESMO, European Society for Medical Oncology; EUSOMA, European Society of Breast Cancer Specialists; JBCS, Japanese Breast Cancer Society; NCCN, National Comprehensive Cancer Network; NICE, National Institute for Health and Care Excellence; SEOM, Sociedad Española de Oncología Médica; TRCR, The Royal College of Radiologists.

^a^ The Program in Evidence‐based Care (PEBC) is supported by Cancer Care Ontario (CCO) and the Ontario Ministry of Health and Long‐Term Care. All work produced by the PEBC are editorially independent from its funding agencies.

### 3.2 Analysis and grading of methodological quality of eligible guideline

The methodological quality of eligible guidelines is different in six domains. Among the six domains of AGREE II, “Clarity of presentation” (74.36 ± 8.50) and “Scope and purpose” (67.31 ± 10.46) were considered as the fields in which eligible guidelines performed best. The domains in which the reviewed eligible guidelines received the lowest mean scores were “Rigor of development” (29.05 ± 24.94) and "Editorial independence" (36.22 ± 21.61). The mean score of the "Stakeholder involvement" and "Applicability” domains was 41.88 ± 26.75 and 39.42 ± 16.13, respectively.

In terms of the overall assessment, guidelines were divided into three levels. Two guidelines developed by NICE^35^ and DGGG & DKG^47^ were classified to be recommended. Only one guideline developed by ARG^21^ was lower than 30 and classified to be not recommended. The remaining guidelines were recommended with modifications. Table 2 lists the results of each domain. The ICC of each CPG AGREE II score among the four reviewers in the study is shown in Table 3, and ranges from 0.694 to 0.984, considerable to outstanding.

**TABLE 2:** Standardized scores of guidelines by AGREE II instrument

Guideline organization, Year	Scope and purpose	Stakeholder involvement	Rigor of development	Clarity of presentation	Applicability	Editorial independence	Overall Assessment	
NICE,2019	83.33	94.44	60.00	75.00	54.17	45.83	65.87	R
CCO, 2018	77.78	2.78	71.88	75.00	43.75	33.33	52.52	RM
TRCR, 2019	52.78	16.67	25.00	72.22	37.50	20.83	35.94	RM
NCCN,2017	69.44	38.89	0.00	63.89	33.33	50.00	36.11	RM
ARG,2007	69.44	16.67	5.21	72.22	16.67	0.00	25.26	NR
CEWG,2018	61.11	44.44	19.79	58.33	75.00	41.67	49.39	RM
SEOM, 2014	58.33	38.89	19.79	86.11	22.92	66.67	41.93	RM
ACR, 2018	66.67	30.56	17.71	72.22	50.00	66.67	46.44	RM
ESMO,2016	55.56	27.78	0.00	75.00	54.17	50.00	39.58	RM
EUSOMA,2020	72.22	33.33	23.96	77.78	29.17	29.17	39.84	RM
ACS,2007	52.78	66.67	35.42	66.67	37.50	0.00	41.49	RM
JBCS, 2019	75.00	47.22	25.00	86.11	20.83	20.83	40.10	RM
DGGG & DKG, 2017	80.56	86.11	73.96	86.11	37.50	45.83	65.19	R
Mean±SD	67.31±10.46	41.88±26.75	29.05±24.94	74.36±8.50	39.42±16.13	36.22±21.61		

Abbreviations: NR, not recommended; R recommended; RM, recommended with modifications.

**TABLE 3:** Intraclass correlation coefficients for each CPG AGREE score

Guideline	Intraclass correlation coefficients	Degree of agreement
NICE,2019	0.743	Considerable
CCO, 2018	0.725	Considerable
TRCR, 2019	0.694	Considerable
NCCN,2017	0.784	Considerable
ARG,2007	0.984	Very good
CEWG,2018	0.905	Very good
SEOM, 2014	0.758	Considerable
ACR, 2018	0.837	Very good
ESMO,2016	0.96	Very good
EUSOMA,2017	0.734	Considerable
ACS,2007	0.879	Very good
JBCS, 2019	0.711	Considerable
DGGG & DKG, 2017	0.843	Very good

Note

ICC <0.20, poor; 0.21–0.40, fair; 0.41–0.60, moderate; 0.61–0.80, considerable; 0.81–1.00, very good.

### 3.3 Level of evidence and strength of recommendation

Five of the guidelines (38.46%) we eventually included used five grade systems to rate the evidence and strength of recommendations. The grading system of the five guidelines is self‐designated, among which the grading system of SEOM^41^ is based on GRADE (Grading of Recommendations Assessment, Development, and Evaluation) system, the grading system of DGGG & DKG is based on OCEBM (Oxford Centre for Evidence‐based Medicine) system, and the remaining three guidelines^38^, ^44^, ^46^ are divided into overall grades for recommendations based on different types of evidence. In different grading systems, the details about the level of evidence and the strength of recommendations are very different. All the information are shown in Table 4.

**TABLE 4:** Grading systems used in included guidelines

Grading systems	Details of evidence and recommendation		Number of guidelines	Guideline organization
	Level of evidence	Strength of recommendation		
NCCN^a^	1, 2A, 2B, 3	―	1	NCCN, 2017
EUSOMA^a^	―	1A, 1B, 1C, 2A, AB, 2C	1	EUSOMA, 2020
SEOM^b^	1,2	A, B, C, D	1	SEOM, 2014
DGGG & DKG^c^	1a, 1b, 1c, 2a, 2b, 2c,3a, 3b, 4,5	A,B,O	1	DGGG & DKG, 2017
JBCS	2, 3	moderate, very weak,weak	1	JBCS, 2019

^a^ Set by themselves based on type of evidence.

^b^ Set by themselves based on GRADE.

^c^ Set by themselves based on Oxford Centre for Evidence‐based Medicine.

### 3.4 Recommendations in eligible guidelines

Table 5 summarizes 54 recommendations from 13 guidelines for screening with pathogenic mutations in hereditary breast cancer predisposition genes, and the evidence basis of the recommendations, the level of evidence, and the strength of recommendations can be seen in the Supplemental. NICE^35^ and ESMO^43^ had the maximum of 17 recommendations (31.48%). Other guidelines had fewer relevant recommendations. The object of the recommendations mainly was the population of BRCA1 or BRCA2 mutation, followed by TP53, ATM, CDH1, CHEK2, NF1, PALB2, NBN, and STK11. Screening methods mainly included magnetic resonance imaging (MRI) and mammography. Although all guidelines recommended screening method in groups with different pathogenic mutations in hereditary breast cancer predisposition genes, the details of these recommendations were inconsistent. Most of the recommendations provided the detailed reports on how patients were screened. Nevertheless, only four recommendations reported the evidence basis for that recommendation in the guideline.

**TABLE 5:** Recommedations of breast cancer due to hereditary predisposition for screening

Genes	Guidelines	Recommendation		
		Who	When	How
BRCA	NICE	BRCA1 or BRCA2 mutation	aged 20–29 years/aged 30–49 years//aged 30–69 years///aged 70 years and over	do not offer MRI/annual MRI//annual mammographic///mammography
	NICE	BRCA1 or BRCA2 mutation^a^	aged 50–69 years	do not offer MRI
	SEOM	BRCA1 or BRCA2 mutation	NR	annual mammography and breast MRI screening
	JBCS	BRCA1 or BRCA2 mutation	NR	contrast‐enhanced breast MRI screening as an adjunct to mammography
	DGGG & DKG, ARG, CEWG	BRCA1 or BRCA2 mutation	NR	MRI
	ACS	BRCA mutation/First‐degree relative of BRCA carrier, but untested	NR	annual MRI
	EUSOMA	BRCA 1/2 mutation carriers other genes (e.g. p53, PALB2, CHEK2, ATM) mutation carriers	NR	annual MRI and mammography with or without ultrasound
	SEOM	Male BRCA2 carriers	starting at age 40	mammography
	ACR	BRCA1 carriers^c^	starting at age 40	mammography
	CEWG	First‐degree female relative with confirmed BRCA1/2 deleterious mutations	NR	genetic testing
	NICE	BRCA1 or BRCA2 mutation, or do not have a TP53 mutation	aged 50–69 years/aged 70 years and over	annual mammographic/mammography
High risk^b^	NICE	High risk of breast cancer^b^	aged 30–59 years	annual mammographic
	NICE	High risk of breast cancer^b^	aged 60 years and over	mammography
	NICE	High risk of breast cancer^b^	any age	Do not offer MRI
TP53	NICE	Without TP53 mutation^a^	aged 50 years and over	Do not offer MRI
	NICE	TP53 mutation	aged 20–69 years	annual MRI
	NICE	TP53 mutation	NR	Do not offer mammographic
	TRCR	TP53 mutation carriers and A‐T (ataxia telangiectasia) homozygotes	NR	Mammography should be avoided
	ESMO	Li Fraumeni Syndrome‐ p53 mutation	age 20 to 25/age 20 to 75	Clinical breast examination every 6 to 12 months/Annual MRI
ATM	NCCN	ATM mutation	starting at age 40	Annual mammogram and consider breast MRI
	ESMO	ATM mutation	NR	annual breast MRI
CDH1	NCCN	CDH1 mutation	starting at age 30	Annual mammogram and consider breast MRI
	ESMO	CDH1 mutation	age 20 to 25/age 20 to 29//age 30 to75	Clinical breast examination every 6 to 12 months/Annual breast MRI//Annual breast MRI and/or mammogram
CHEK2	NCCN	CHEK2 mutation	starting at age 40	Annual mammogram and consider breast MRI
	ESMO	CHEK2 mutation	age 20 to 25/age 20 to 29//age 30 to75	Clinical breast examination every 6 to 12 months/Annual breast MRI//Annual breast MRI and/or mammogram
NF1	NCCN	NF1 mutation	starting at age 30/ages 30–50 years	Annual mammogram/MRI
PALB2	NCCN	PALB2 mutation	starting at age 30	Annual mammogram and consider breast MRI
	ESMO	PALB2 mutation	age 20 to 25/age 20 to 29//age 30 to75	Clinical breast examination every 6 to 12 months/Annual breast MRI//Annual breast MRI and/or mammogram
PTEN	ESMO	PTEN/Cowden Syndrome	age 20 to 25/age 30 to 75	Clinical breast examination every 6 to 12 months/Annual breast MRI and/or mammogram
STK11	ESMO	STK11 mutation (Peutz–Jeghers Syndrome)	age 20 to 25/age 20 to 29//age 30 to75	Clinical breast examination every 6 to 12 months/Annual breast MRI//Annual breast MRI and/or mammogram
NBN	NCCN	NBN mutation	starting at age 40	Annual mammogram and consider breast MRI

^a^ unless mammography has shown a dense breast pattern.

^b^ Lifetime risk of developing breast cancer is at least 30%. (High‐risk group includes rare conditions that carry an increased risk of breast cancer, such as Peutz‐Jegher syndrome, (STK11), Cowden (PTEN), familial diffuse) gastric cancer (E‐Cadherin).

^c^ if they are imaged yearly with contrast‐enhanced breast MRI starting at age 25.

Of the 54 recommendations in the 13 included guidelines, 11 guidelines contained 20 recommendations (37.04%) for BRCA, except for one^42^ recommendation for BRCA1. The remaining recommendations are for BRCA1 and BRCA2. ACR recommended starting mammography screening at age 40 for this group. However, it did not report the frequency of screening when the screening was terminated. Of all the guidelines, only SEOM^41^ recommended to use mammography screening in men aged 40 for BRCA2 genetic mutation. There were also two guidelines^40^, ^45^ that made recommendations for the first‐degree relative BRCA carriers, CEWG^40^ recommended genetic testing, and ACS^45^ recommended annual MRI.

For BRCA carriers, we summarized the recommendations and found that most of the recommendations are focused on MRI and mammography. NICE,^35^ CCO,^36^ ARG,^39^ CEWG,^40^ EUSOMA,^44^ ACS,^45^ JBCS,^46^ and DGGG & DKG^47^ recommended to perform MRI screening for BRCA1 or BRCA2 gene mutation carriers, but the recommendations of each guideline were different in the details. NICE^35^ recommended annual MRI screening in the 30–45 age group. CCO^36^ recommended MRI and mammography, but there were no restrictions on the age of screening. ARG,^39^ CEWG,^40^ and DGGG & DKG^47^ all recommended MRI, but there were also no restrictions on the age or frequency of screening subjects. EUSOMA^44^ recommended annual MRI and mammography with or without ultrasound, ACS^45^ recommended annual MRI, JBCS^46^ recommended contrast‐enhanced breast MRI screening as an adjunct to mammography, and SEOM^41^ recommended annual mammography and breast MRI screening. These guidelines all recommended MRI, but the specific screening methods were not the same. For BRCA1 or BRCA2 mutation carriers, NICE^35^ recommends annual mammograms for those aged from 30 to 69 years old, while it does not recommend MRI screening for those aged 20–29 and 50–69 years old.

There were three guidelines with eight recommendations concerning TP53 gene (P53) mutations. All the recommendations were consistent without conflict. MRI was recommended, and mammography was not recommended. NICE^35^ had five recommendations for TP53 gene mutations, and MRI was not recommended for people aged 50 years old and over and had no TP53 gene mutations (unless mammography has shown a dense breast pattern). NICE^35^ recommended annual MRI screening for TP53 mutations in people aged between 20 and 69 years old. Both NICE and TRCR did not recommend screening such people with mammography. ESMO^43^ recommended clinical breast examination every 6–12 months for Li–Fraumeni Syndrome‐p53 mutation aged 20–25 years old, and annual MRI between 20 and 75 years old.

Regarding ATM gene mutations, NCCN^38^ and ESMO^43^ recommended annual MRI, and NCCN^38^ also recommended mammography screening for people aged 40, while ESMO^43^ only recommended annual MRI screening with no age limit. As for CDH1 mutation, CHEK2, or PALB2 mutation, MRI or mammography screening was recommended.^38^, ^43^ In conclusion, we found that the main screening methods were focused on mammography or MRI, and the recommendations were consistent.

## 4 DISCUSSION

The occurrence and development of breast cancer are complex biological processes, involving genetic factors, nongenetic factors, and their interaction. Early screening for breast cancer has always been a research hotspot. Gene mutation is closely related to breast cancer, and breast cancer screening for people with gene mutation can reduce the mortality rate, which has been recognized by major international organizations in the field.^7^, ^48^

To the best of our knowledge, this review represented the largest and most comprehensive assessment and summary of the screening guidelines and recommendations on the genetic mutation of breast cancer that is conducted to date. We also tried AGREE II tool to evaluate the quality of the included guidelines. A total of 13 guidelines that met the inclusion and exclusion criteria were included. "Rigor of development" scored the lowest, while "Clarity of Presentation" scored the highest. Furthermore, only two guidelines^35^, ^47^ (15.38%) scored more than 60 and were recommended. However, we discovered that most guidelines only describe the level of evidence that supports the recommendations, and the strength and grading of recommendations vary from different guidelines. It will somewhat impede the implementation of the guidelines and the communication among different guideline development teams.^49^ A standardized grading system is necessary to provide clear information about the level of evidence and the strength of recommendations. Most importantly, screening guidelines for people with pathogenic mutations in hereditary breast cancer predisposition genes should focus more on evidence. Notably, 54 recommendations were included, and only four (7%) recommendations identified the evidence base.

The report stated that receiving diagnostic radiation before the age of 30 years old is associated with the increased risk of breast cancer in BRCA1 or BRCA2 carriers, and the dose level is much lower than the increased risk in other groups exposed to radiation.^50^ The guidelines we included do not mention screening before the age of 30 for this group. Only NICE^35^ recommended that BRCA1 or BRCA2 mutation people between the age of 20 and 29 do not undergo MRI screening. As for the recommendations in the BRCA mutation, guidelines were focused on mammography and MRI, except for specific age groups where MRI screening was not recommended. Studies have shown that mammography adds only a small amount of cancer detection to BRCA1 mutation carriers under 40 years old if screening with MRI regularly,^51^, ^52^, ^53^ while BRCA2 mutation carriers benefit from mammography and MRI, because more cancers are found only through mammography.^54^, ^55^ In summary, for this population, screening methods focus on mammography and MRI or their combination.

The incidence of TP53 mutations is low, but it has great clinical significance. TP53 mutations are associated with breast cancer, and 95% of these mutations cause breast cancer, and many mutations occur at an early age.^56^ Studies have revealed that radiation exposure can lead to the higher incidence of secondary tumors in carriers of TP53 mutation.^57^ For the population with TP53 mutation, the recommendations of the guidelines are relatively clear and consistent.^35^, ^37^, ^43^ It is believed that mammography screening should not be selected for this group of people and MRI screening should be conducted every year for people aged from 20 to 69 or 70. In a recent multicore and randomized controlled study, MRI screening‐detected breast cancer is earlier than mammography in women with TP53 mutations.^58^ In general, the screening method for this group is relatively clear, but the lack of evidence in the guidelines hinders users’ understanding to some extent.

For some other gene mutations, such as ATM, CDH1, CHEK2, NF1, PALB2, etc., based on the screening age and screening method, we have extracted the recommendations into the table. Recommendations for these genes are relatively few, but their clinical importance cannot be ignored. Only three guidelines cover these genes^38^, ^43^, ^44^ It is suggested that more high‐quality studies should be carried out in the future as the evidence basis for the guidelines to increase the credibility of the guidelines.

Overall, recommendations for people with different pathogenic mutations in hereditary breast cancer predisposition genes are mostly consistent, and only a few details are unclear. In the future breast cancer screening guidelines for gene mutations, attentions should be paid to the report of the evidence basis and the unification of the grading system, and the report of the frequency and age of screening. This study summarizes the recommendations and will help clinical decision‐makers and patients to choose screening methods.

### 4.1 Existing challenges in screening for breast cancer due to hereditary predisposition

Early detection of carriers of pathogenic mutations in breast cancer susceptibility genes before the onset of breast cancer is significant for a successful breast cancer screening. However, it is estimated that the identity of the carriers of most pathogenic mutations in hereditary breast cancer predisposition genes still remains unclear.^59^, ^60^ Many screening tools were used to assess the likelihood of pathogenicity‐related gene mutations. USPSTF has confirmed that the main tools are as following: BRCAPRO,^61^ Ontario Family History Risk Assessment Tool,^62^ Pedigree Assessment Tool,^63^ Manchester Scoring System,^64^ and 7‐Question Family History Screen (FHS‐7).^65^ More efforts are needed to effectively screen carriers of disease‐causing mutations in breast cancer susceptibility genes.

### 4.2 Strengths and limitations

This review has several strengths. First, this is the first study to analyze the screening recommendation of breast cancer due to hereditary predisposition. Second, we conducted a comprehensive search on global breast cancer guidelines. Third, we used the AGREE II instrument to assess the methodology quality, which partially reflected the quality of the guidelines. Finally, all of our authors are professionally trained and have rich experience in the evaluation of guidelines to ensure reliability.

On the other hand, our research also has some limitations. Few of the guidelines clearly described the evidence basis of the recommendations, and most of the guidelines were inconsistent in the grading system, which made it difficult for us to integrate recommendations. Moreover, the methodological quality of the guidelines represents the credibility of a part of the guidelines, but it cannot decide whether the guidelines should be recommended.

## 5 CONCLUSIONS

This systematic review reports a broad and comprehensive summary of the recommendations of the latest international screening guidelines for genetic susceptibility to breast cancer. Among them, most recommendations were for BRCA mutations. Besides, the screening recommendations in different guidelines are generally consistent. The mammography and MRI were frequently recommended in the eligible guideline. Moreover, the overall quality of the 13 eligible guidelines was divergent. There is much room for quality improvement, especially in "Rigor of the development." The search process should be improved and the basis of recommendations should be reported, and the evidence grade system should be standardized (e.g., using GRADE) to provide more powerful supporting evidence for guideline users, which is more conducive to the understanding and dissemination of the guidelines. In this study, recommendations were sorted into three aspects, namely "Who," "When," and "How", to offer better guidance for clinicians, health‐care practitioners, and patients.

## CONFLICT OF INTERESTS

The authors declare no conflict of interest.

## Ethical Approval Statement

Not applicable

## Supporting information

Table S1

## Data Availability

Data sharing not applicable to this article as no datasets were generated or analyzed during the current study.
